# Supplementary material for: Sexual assault and abuse committed against family members: An analysis of 1342 legal outcomes and their motivations
Source: PLoS One. 2021 Jun 29;16(6):e0253980. doi: 10.1371/journal.pone.0253980 (PMC8241090; doi:10.1371/journal.pone.0253980)
Supplement: S1 Table — (DOCX) [file pone.0253980.s001.docx]

**S1 Table. Distribution of victims according to nationality and occupation.**

| **VICTIMS** | **Conviction** | | | | | |  | **Acquittal** | | | | | |
| --- | --- | --- | --- | --- | --- | --- | --- | --- | --- | --- | --- | --- | --- |
|  | **Total*** | | **SV** | | **DV** | |  | **Total*** | | **SV** | | **DV** | |
|  | **N** | **%** | **n** | **%** | **n** | **%** |  | **n** | **%** | **n** | **%** | **n** | **%** |
| **Nationality** |  |  |  |  |  |  |  |  |  |  |  |  |  |
| Italian | 551 | 61.9 | 159 | 61.4 | 357 | 64.0 |  | 263 | 58.2 | 59 | 51.3 | 189 | 59.6 |
| European (Non-Italian) | 82 | 9.2 | 20 | 7.7 | 54 | 9.7 |  | 44 | 9.7 | 16 | 13.9 | 28 | 8.8 |
| African | 54 | 6.1 | 11 | 4.3 | 35 | 6.3 |  | 20 | 4.4 | 5 | 4.4 | 14 | 4.4 |
| Sudamerican | 52 | 5.8 | 16 | 6.2 | 26 | 4.7 |  | 47 | 10.4 | 9 | 7.8 | 37 | 11.7 |
| Asian | 55 | 6.2 | 11 | 4.3 | 36 | 6.5 |  | 26 | 5.8 | 6 | 5.2 | 20 | 6.3 |
| Information Missing | 96 | 10.8 | 42 | 16.2 | 50 | 9.0 |  | 52 | 11.5 | 20 | 17.4 | 29 | 9.2 |
| **Occupation** |  |  |  |  |  |  |  |  |  |  |  |  |  |
| Unemployed | 27 | 3.0 | 5 | 1.9 | 14 | 2.5 |  | 20 | 4.4 | 8 | 7.0 | 12 | 3.8 |
| Employee | 87 | 9.8 | 37 | 14.3 | 41 | 7.4 |  | 38 | 8.4 | 14 | 12.2 | 17 | 5.4 |
| Freelance | 7 | 0.8 | 2 | 0.8 | 3 | 0.5 |  | 2 | 0.4 | 0 | 0 | 2 | 0.6 |
| Housewife | 21 | 2.4 | 6 | 2.3 | 12 | 2.2 |  | 5 | 1.1 | 2 | 1.7 | 3 | 1.0 |
| Another job | 127 | 14.3 | 62 | 23.9 | 55 | 9.9 |  | 69 | 15.3 | 23 | 20.0 | 45 | 14.2 |
| Information Missing | 621 | 69.8 | 147 | 56.8 | 433 | 77.6 |  | 318 | 70.4 | 68 | 59.1 | 238 | 75.1 |

Total sample include sexual violence crimes (SV), abuses against family members or cohabitants crimes (DV) and cases concerning both of them.
