# Supplementary material for: Sexual assault and abuse committed against family members: An analysis of 1342 legal outcomes and their motivations
Source: PLoS One. 2021 Jun 29;16(6):e0253980. doi: 10.1371/journal.pone.0253980 (PMC8241090; doi:10.1371/journal.pone.0253980)
Supplement: S2 Table — (DOCX) [file pone.0253980.s002.docx]

**S2 Table. Distribution of defendants according to nationality.**

| **DEFENDANTS** | **Conviction** | | | | | |  | **Acquittal** | | | | | | |
| --- | --- | --- | --- | --- | --- | --- | --- | --- | --- | --- | --- | --- | --- | --- |
|  | **Total*** | | **SV** | | **DV** | |  | **Total*** | | **SV** | | **DV** | | |
|  | **n** | **%** | **n** | **%** | **n** | **%** |  | **n** | **%** | **n** | **%** | **n** | **%** |  |
| **Nationality** |  |  |  |  |  |  |  |  |  |  |  |  |  |  |
| Italian | 495 | 55.6 | 136 | 52.5 | 323 | 57.9 |  | 281 | 62.2 | 62 | 53.9 | 204 | 64.4 |  |
| European | 89 | 10.0 | 22 | 8.5 | 58 | 10.4 |  | 41 | 9.1 | 14 | 12.2 | 26 | 8.2 |  |
| African | 135 | 15.2 | 49 | 18.9 | 73 | 13.1 |  | 47 | 10.4 | 16 | 13.9 | 29 | 9.2 |  |
| Nord American | 10 | 1.1 | 2 | 0.8 | 8 | 1.4 |  | 2 | 0.4 | 1 | 0.9 | 1 | 0.3 |  |
| Sudamerican | 70 | 7.9 | 23 | 8.9 | 40 | 7.2 |  | 48 | 10.6 | 11 | 9.6 | 36 | 11.4 |  |
| Asian | 78 | 8.8 | 22 | 8.5 | 48 | 8.6 |  | 28 | 6.2 | 9 | 7.8 | 19 | 6.0 |  |
| Information Missing | 13 | 1.5 | 5 | 1.9 | 8 | 1.4 |  | 5 | 1.1 | 2 | 1.7 | 2 | 0.6 |  |

Total sample include sexual violence crimes (SV), abuses against family members or cohabitants crimes (DV) and cases concerning both of them.
