# Supplementary material for: Sexual assault and abuse committed against family members: An analysis of 1342 legal outcomes and their motivations
Source: PLoS One. 2021 Jun 29;16(6):e0253980. doi: 10.1371/journal.pone.0253980 (PMC8241090; doi:10.1371/journal.pone.0253980)
Supplement: S3 Table — (DOCX) [file pone.0253980.s003.docx]

**S3 Table. Distribution of relationship between victims and defendants.**

| **Bond victims/defendants** | **Conviction** | | | | | |  | **Acquittal** | | | | | |
| --- | --- | --- | --- | --- | --- | --- | --- | --- | --- | --- | --- | --- | --- |
|  | **Total*** | | **SV** | | **DV** | |  | **Total*** | | **SV** | | **DV** | |
|  | **n** | **%** | **n** | **%** | **n** | **%** |  | **n** | **%** | **n** | **%** | **n** | **%** |
| Husband | 277 | 31.1 | 16 | 6.2 | 225 | 40.3 |  | 136 | 30.1 | 13 | 11.3 | 116 | 36.6 |
| Ex-husband | 14 | 1.6 | 1 | 0.4 | 13 | 2.3 |  | 9 | 2.0 | 2 | 1.7 | 7 | 2.2 |
| Cohabitant Partner | 149 | 16.7 | 17 | 6.6 | 116 | 20.8 |  | 78 | 17.3 | 9 | 7.8 | 63 | 19.9 |
| Ex- cohabitant partner | 8 | 0.9 | 4 | 1.5 | 3 | 0.5 |  | 9 | 2.0 | 2 | 1.7 | 7 | 2.2 |
| Non-cohabitant partner | 10 | 1.1 | 5 | 1.9 | 3 | 0.5 |  | 5 | 1.1 | 1 | 0.9 | 4 | 1.3 |
| Ex non-cohabitant partner | 4 | 0.5 | 3 | 1.2 | 1 | 0.2 |  | 5 | 1.1 | 4 | 3.5 | 1 | 0.3 |
| Other family member | 205 | 23.0 | 22 | 8.5 | 173 | 31.0 |  | 125 | 27.7 | 10 | 8.7 | 112 | 35.3 |
| Friend | 119 | 13.4 | 95 | 36.7 | 18 | 3.2 |  | 56 | 12.4 | 46 | 40.0 | 6 | 1.9 |
| Not known | 104 | 11.7 | 96 | 37.1 | 6 | 1.1 |  | 29 | 6.4 | 28 | 24.4 | 1 | 0.3 |

Total sample include sexual violence crimes (SV), abuses against family members or cohabitants crimes (DV) and cases concerning both of them.
