# Supplementary material for: Sexual assault and abuse committed against family members: An analysis of 1342 legal outcomes and their motivations
Source: PLoS One. 2021 Jun 29;16(6):e0253980. doi: 10.1371/journal.pone.0253980 (PMC8241090; doi:10.1371/journal.pone.0253980)
Supplement: Attachment — Submitted filename: Response to Reviewers.docx [file pone.0253980.s004.docx]

Dear editor,

We would like to thank you and the reviewer for the attention reserved to our paper.

Following advice and comments proposed by the reviewer, changes were made, specified in detail below:

Journal Requirements

*2. We note that you have included the phrase “data not shown” in your manuscript. Unfortunately, this does not meet our data sharing requirements. PLOS does not permit references to inaccessible data. We require that authors provide all relevant data within the paper, Supporting Information files, or in an acceptable, public repository. Please add a citation to support this phrase or upload the data that corresponds with these findings to a stable repository (such as Figshare or Dryad) and provide and URLs, DOIs, or accession numbers that may be used to access these data. Or, if the data are not a core part of the research being presented in your study, we ask that you remove the phrase that refers to these data.*

We have included the data in the text.

Reviewer’s comments:

“*To assess the effects of victim characteristics, defendant characteristics, crime characteristics, and medical information on case outcomes, the authors conduct a series of logistic regression analyses addressing each set of independent variables. Typically, in a study like this, the analysis would include all of the independent variables in the model (either entering the variables in blocks or entering them all initially). Given that the authors' approach differs from this, they should provide some justification for not including all of the factors in the final model.”*

As required from the referee, we applied the backward stepwise regression method and reported the adjusted odds ratios and the relative 95% confidence interval of the final multivariate logistic regression model concerning the overall sample. To allow the comparison of the results, we include in the analyses of the two subgroups (sexual violence and domestic violence) the same variables of the final logistic regression model of the overall sample. Results have been modified accordingly.
